# Is there a link between blastomere contact surfaces of day 3 embryos and live birth rate?

**DOI:** 10.1186/1477-7827-10-78

**Published:** 2012-09-10

**Authors:** Goedele Paternot, Mathias Spiessens, Dimitri Verstreken, Johan Van Bauwel, Sophie Debrock, Thomas D’Hooghe, Carl Spiessens

**Affiliations:** 1 Leuven University Fertility Center, UZ Leuven, Gasthuisberg, Campus gasthuisberg, Leuven, Belgium; 2 Lessius-Mechelen, Campus De Nayer, Sint-Katelijne-Waver, Belgium

**Keywords:** Blastomere contact surfaces, Day 3 embryos, Life birth, Compaction, Single embryo transfer

## Abstract

**Background:**

Cell-cell communication and adhesion are essential for the compaction process of early stage embryos. The aim of this study was to develop a non-invasive objective calculation system of embryo compaction in order to test the hypothesis that embryos with a larger mean contact surface result in a higher live birth rate compared to embryos with a lower mean contact surface.

**Methods:**

Multilevel images of 474 embryos transferred on day 3 were evaluated by the Cellify software. This software calculates the contact surfaces between the blastomeres. The primary outcome of this study was live birth. An ideal range of contact surface was determined and the positive and negative predictive value, the sensitivity, the specificity and the area under the curve for this new characteristic were calculated.

**Results:**

In total, 115 (24%) transferred embryos resulted in a live birth. Selection of an embryo for transfer on its mean contact surface could predict live birth with a high sensitivity (80%) and high negative predicting value (83%) but with a low positive predictive value (27%), a low specificity (31%) and low area under the ROC curve (0.56). The mean contact surface of embryos cultured in a single medium was significantly higher compared to the mean contact surface of embryos cultured in a sequential medium (p = 0.0003).

**Conclusions:**

Neither the mean contact surface nor the number of contact surfaces of a day 3 embryo had an additional value in the prediction of live birth. The type of culture medium, however, had an impact on the contact surface of an embryo. Embryos cultured in a single medium had a significant larger contact surface compared to embryos cultured in the sequential medium.

## Background

Cell-cell interactions and subsequent communication are essential for the compaction process in early stage embryos and the subsequent pre-implantation development [1-3]. The expression of cell adhesion molecules during pre-implantation embryo development is therefore crucial at different stages. It has been shown that the human embryo expresses different types of adhesion molecules [4,5] like E-cadherin adhesion molecules, gap junctions, desmosomes and tight junction complexes.

Cadherins are calcium-dependent transmembrane glycoproteins that play an important role in cell polarity, cell signaling and cell adhesion [6]. In mice, E-cadherin has been reported to be the first cadherin expressed in embryo development, after embryonic genome activation [7]. In E-cadherin knock-out mice, embryos can compact based on the maternally inherited protein but normal trophectoderm differentiation is absent (due to absence of subsequent E-cadherin expression) which is lethal for the embryo [8,9].

The distribution pattern of E-cadherin is crucial in the pre-implantation embryo [10]. It is stage-dependent and disturbances in the distribution occur in embryos with cleavage abnormalities and result in loss of embryonic viability [10]. During the first three days of development, the protein appears to be cytoplasmic and is concentrated in the non-contact regions of cells in morphologically normal embryos. E-cadherin is relocated to cell-cell contact regions on day 4 and after differentiation of the embryonic cells, the distribution of E-cadherin is cytoplasmic in ICM cells and located at cell-cell contacts in trophectoderm cells [10]. Since an abnormal staining pattern was also found in morphologically normal embryos this indicates that morphological evaluation, based on currently used characteristics, is insufficient to define the viability of embryos [10].

Gap junctions are intercellular structures that are responsible for the electrical and chemical communication between two cells [11]. They can be found as early as the 4-cell stage [12]. Morphological normal embryos, however, can have a deficit in the number of gap junctions [12].

Other cell-adhesion molecules found in the pre-implantation embryo are desmosomes [13,14] and tight junctions complexes [15,16]. Desmosome junctions mediate intercellular adhesion by transmembrane molecules (desmocollin and desmogelin). Tight junctions consists of multi-protein complexes, responsible for cell signaling and maintenance of cell polarity [15,16]. Both types of cell adhesion molecules are formed at the 32-cell stage at the onset of blastocoel cavitation [17]. Data on mRNA expression profiles for both types of cell junction molecules showed that reduced mRNA availability due to reduced transcription or increased turnover, may impair embryo viability in vitro. In addition, the expression of these molecules has been linked to amino acid turnover [18]. It was found that the amino acid turnover is related to junction formation and may predict trophectoderm integrity. A relatively high amino acid turnover at compaction and cavitation stage may be related to embryos with better developmental potential [19].

Currently, cell-cell communication or adhesion are evaluated in early stage embryos as a marker for the compaction process in embryos. Early compaction on day 3 of development is a valuable additional morphological characteristic in the evaluation of embryos since it is strongly associated with implantation potential [20]. Moreover, it was suggested that compacted embryos proved that they were able to activate the embryonic genome and to reach an advanced developmental stage explaining the additive value of this characteristic [21]. Therefore, this characteristic has been included in different scoring systems to determine the embryo with the highest implantation potential [20-22].

In view of the current limitations to evaluate cell-cell adhesion in embryos in clinical practice (2D morphological assessment, invasive assessment), our study was done to develop a new objective and non-invasive method using multilevel images to calculate the contact surfaces between blastomeres of day 3 embryos. We tested the hypothesis that embryos with a larger mean contact surface result in a higher live birth rate compared to embryos with a lower mean contact surface. In addition, we evaluated the effect of two different types of culture medium on the contact surfaces of embryos as it has been reported that modifications of embryo environment can improve junctional communications between cells [12,23].

## Methods

### Patients

A total number of 474 transferred embryos, with a maximum of 8-cells, was evaluated in this study resulting from 474 patients younger than 36 years (30.61 ±3.31) who received a single embryo on day 3 in their first ART cycle. The study was approved by Institutional Review Board of the University Hospitals Leuven (ML4564). Patients were stimulated using the protocol described by Debrock et al. [24]. Patients were excluded when a biopsy for pre-implantation genetic diagnosis was performed or if donor sperm/donor oocytes were used. Patients and cycle characteristics are listed in Table 1. 

**Table 1:** Patients and cycle characteristics

**Patients**	**N = 474**
Female age	30.61 ± 3.31
Cause of subfertility	
● Unexplained	219 (46)
● Tubal	60 (13)
● Ovulation	103 (22)
● Endometriosis	69 (15)
● Implantation	12 (3)
● Other	11 (2)
● Male factor	339 (72)
Cycles	N = 474
● ICSI	189 (39%)
● IVF	288 (61%)
● Oocytes per oocyte retrieval	10.16 ± 4.71
● Mature oocytes per oocyte retrieval	8.84 ± 4.15
● Fertilization rate per oocyte	65 ± 21.6
● Fertilization rate per mature oocyte	74 ±21.3
Clinical outcome	
● Positive βhCG	158 (33)
● Biochemical pregnancy	18 (4)
● Implanted embryos	140 (30)
● Extra uterine sac	2 (0.4)
● Intra uterine sac	138 (29)
● Early miscarriage^a^	18 (4)
● Late miscarriage^b^	4 (1)
● Induced abortion	1 (0)
● Live birth	115 (24)
Embryo characteristics (n = 474)	Total
● ≤ 5 cell stage	46 (10)
● 6-cell stage	47 (10)
● 7-cell stage	102 (22)
● 8- cell stage	279 (59)

Values are mean ± SD or N(%).

^a^defined as a miscarriage up to 12 weeks after embryo transfer.

^b^ defined as a miscarriage between 12 and 20 weeks after embryo transfer.

### ART procedure

The oocytes were retrieved and inseminated as previously described by our group [25]. After oocyte retrieval, the oocytes were washed through four wells each containing 500 μl fertilization medium (sequential medium COOK medium, Sydney IVF fertilization, Sydney IVF, Queensland, Australia or a single medium GM501 medium, Gynemed Lensahn Germany) (37°C, pH 7.25-7.35) under mineral oil. Spermatozoa used for the IVF procedure were prepared using standard density gradient procedures (Isolate, Irvine Scientific, USA). Sperm samples used for ICSI were diluted and were centrifuged two times during 10 minutes (300 g). Standard in-vitro fertilization (IVF)/intracytoplasmic sperm injection (ICSI) procedures were performed 2–6 hours after oocyte retrieval. During the IVF procedure, oocytes were inseminated with 300 000 progressively motile spermatozoa per well (5 oocytes in 0.5 ml). In case of an ICSI cycle, injected oocytes were incubated together in a 20 μl culture medium droplet under oil. On day 1 (16-20 h after insemination/injection) fertilization control took place. Only normally fertilized oocytes (2PN) were cultured individually in a 20 μl droplet of culture medium covered with mineral oil. In total, 1520 fertilized oocytes were cultured in the sequential medium of which 241 embryos were transferred on day 3. A total number of 1430 fertilized oocytes, of which 233 embryos were transferred on day 3, were cultured in the single medium.

### Embryo evaluation

Image sequences were recorded of each embryo on day 1 (16-20 h after insemination/injection), day 2 (41 h-44 h after insemination/injection) and day 3 (66-71 h after insemination/injection) using the computer assisted scoring system. The semi-automatically embryo quality assessment, using this system, has been recently described by our group [26]. Based on the 26 sequential images (Z-stacks) of the same embryo, the system calculates the number of blastomeres, the total cytoplasmic reduction (which can be interpreted as the degree of fragmentation) and the size of blastomeres. In addition, the embryos were evaluated using the manual scoring system of the Leuven University Fertility Centre. This embryo evaluation is based on the assessment by an embryologist who evaluated the number and size of blastomeres and the degree of fragmentation. On day 3 of embryo development the best embryo was chosen for transfer based on the manual scoring system.

### Evaluation of contact surfaces

**Figure 1:**
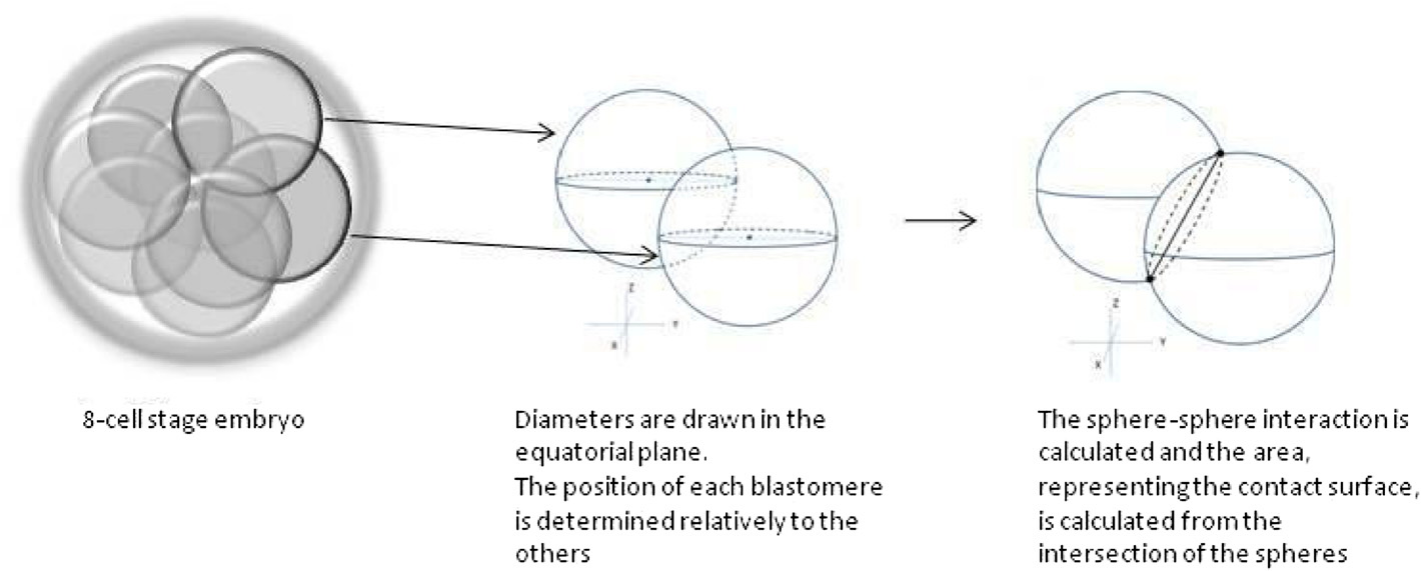
Animation of the contacts surface measurement between adjacent blastomeres by the Cellify software program.

To evaluate the blastomere contact surfaces between blastomeres, the multilevel images were uploaded in a newly developed software package (Cellify).This software allows to calculate the contact surface between adjacent blastomeres of embryos up to the 8-cell stage (Figure 1). The software is based on different tiff files with different foci (=multilevel images). In each blastomere, perpendicular diameters are drawn manually in the equatorial plane. Based on the intersection point of these diameters, the system determines the position of each blastomere relatively to the others. Blastomeres are represented as spheres of which the volume is calculated using the drawn diameters. The sphere-sphere interaction is calculated and the area, representing the contact surface, is calculated from the intersection of the spheres (Figure 1).

### Statistics

The primary outcome of this study was live birth. In order to determine the range of the “ideal contact surface” of an embryo, the mean and standard deviation of the contact surface of those embryos whose transfer resulted in a live birth, were calculated. In a next step, based on this “ideal range”, the positive and negative predictive value, the sensitivity, the specificity and the area under the curve were evaluated. Finally, simple and multiple logistic regression analyses were performed using live birth rate as endpoint. The number of contact points, the contact surface and the number of blastomeres on day 3 were included in the analyses. To evaluate the impact of the type of culture medium on the contact surfaces, a t-test was performed with a significance level of 0.05.

## Results

In total, 115 (24%) transferred embryos resulted in a live birth. The mean contact surface of these embryos was 18945.5 pixels (±5670.9). Based on these data, the “ideal” range of contact surfaces was defined based on the mean ± 1SD (13274.6 pixels-24616.4 pixels) (Table 2).

**Table 2:** Mean and SD of contact surfaces of embryos resulting in a live birth

Number of embryos resulting in live birth	115 (24)
Mean contact surface (±SD) of the total group of transferred embryos	19708.2. (±7238.53) pixels
Mean contact surface (±SD) of embryos resulting in live birth	18945.5 (±5670.9) pixels
Ideal range	13274.6-24616.4 pixels
Total number of embryos within ideal range	341
Total number of embryos outside ideal range	133
Positive predictive value	27%
Negative predictive value	83%
Sensitivity	80%
Specificity	31%
Area under the curve	0.56

The total group of embryos were subdivided in two groups: embryos with a mean contact surface within the “ideal” range (mean contact surface +/− 1 standard deviation, based on the embryos resulted in a life birth) and embryos with a mean contact surface outside the defined range (embryos with a lower or higher contact surface). In order to evaluate this new morphological characteristic, the positive and negative predictive value, the sensitivity and specificity and the area under the curve was calculated (Table 2).

The results indicated that the selection of an embryo for transfer on its mean contact surface could predict live birth with a high sensitivity (80%) and high negative predicting value (83%) but with a low positive predictive value (27%), a low specificity (31%) and low area under the ROC curve (0.56) (Table 2).

In a simple logistic regression using live birth as endpoint, the impact of the contact surface and the impact of the number of contact points were evaluated (Table 3). Both characteristics had a significant impact on the live birth rate of the embryo. However, when a multiple regression analysis was performed including the number of blastomeres on day 3, both characteristics failed to be significant (Table 3).

**Table 3:** Results of simple and multiple logistic regression analyses using live birth as endpoint

**Regression analysis**	**p-value**
Simple logistic regression	
● Mean contact surface	<0.001
● Number of contact points	<0.001
Multiple regression analysis	
● Mean contact surface	NS
● Number of contact points	NS
● Number of blastomeres	<0.001

In conclusion, the mean contact surface nor the number of contact surfaces of an embryo had an additional value in the prediction of live birth.

In order to evaluate a possible impact of culture conditions on the contact surfaces of early stage embryos, a comparison was made between the transferred embryos cultured in a sequential medium (n = 241) and embryos cultured in a single medium (n = 233). No significant differences were found regarding live birth rate between both groups of embryos (sequential medium: 25% (61/241); single medium: 23% (54/233)). Although, a significant difference was found when both groups of embryos were compared based on the mean contact surface. The contact surface of embryos cultured in the single medium was significantly higher (20928 pixels ±6878) compared to the mean contact surface of embryos cultured in the sequential medium (18528 pixels ±7396) (p = 0.0003).

## Discussion

Currently, cell-adhesion or compaction stage can only be evaluated in human embryos using subjective or invasive techniques [20-22,27]. Evaluation of compaction in early stage embryos by an embryologist has been described, but intra and inter-observer variability is unknown. Other approaches to evaluate the cell-cell communication or the grade of compaction are invasive and therefore not applicable in daily routine: staining embryos (e.g. the combination of laser scanning microscopy and fluorescent markers) for the detection of specific adhesion molecules makes the embryos unavailable for transfer [12,18,28-31].

In this prospective study, a novel, non-invasive and therefore clinically applicable quantitative assessment of contact surfaces between blastomeres in day 3 embryos was used in order to test the hypothesis that embryos with a larger contact surface between blastomeres had a higher viability. An important strength of this study is the use of a non-invasive method to evaluate the contact surfaces which is in contrast to other studies evaluating the cell-cell communication using invasive staining techniques [12,18,28,29,31]. Using a non-invasive method has the advantage to be applicable in daily routine. Moreover, multilevel images were used, which allows the imitation of a microscopic evaluation of the embryo at different foci. In addition, the use of an objective measurement of the contact surfaces makes the design of this study unique. Other studies used a grading system based on the assessment by an embryologist which contains a subjective component in the evaluation of embryo quality [20-22,27]. Moreover, single embryo transfers were performed in a selected group of good prognosis patients, which allows direct calculation of clinical relevance.

Although this study has a unique design, it also has some limitations. The analysis of the multilevel images is time consuming since the diameters of every individual blastomere have to be outlined manually. An automatic blastomere identification would reduce the time needed for the evaluation of the contact surfaces. In addition, the accuracy of the measurements of the system could be improved by reducing the distance between the Z-stacks which would result in a more accurate positioning of the blastomeres within the zona pellucida. In addition, the system is limited to the evaluation of embryos with a maximum of 8-cells. Optimization of the software and hardware could solve these issues but the primary goal of this study, test the efficiency of a new morphological characteristic, was achieved.

Whether the mean contact surface reflects the process of compaction remains a question. Compaction takes place between day 3 and day 4. It can be confirmed in a blastocyst culture system in which compaction can be evaluated on day 4. The measurement of the mean contact surface at day 4 represents a major challenge since at the morula stage defining the individual blastomeres becomes more difficult.

Other authors already suspected an influence of culture conditions on the embryo viability [12]. As well known, embryonic arrest in early stage embryos is due to the exposure to suboptimal conditions [12]. As reported previously, the type of culture medium has an impact on the embryo quality and implantation potential [25,32-36]. The results in this study confirmed the influence of the type of culture medium on the embryo quality and viability. The data showed that the mean contact surfaces of embryos cultured in a single medium are significantly higher compared to the contact surfaces of embryos cultured in a sequential medium. Whether this reflects a difference in embryo quality due to a faster development remains to be investigated.

## Conclusions

Future challenges will be the extension of the program software to the evaluation of higher stage embryos, the automation of the blastomere detection and automatic positioning of the blastomeres within the zona pellucida. This will only be possible when a complete 3D image can be constructed using the Z-axis of the embryo. To obtain this, multilevel images are needed from different positions which is technically impossible so far. If these hurdles can be overcome, multilevel images of human embryos will allow embryologists to obtain more objective measurements of embryo characteristics which in turn will improve the reproducibility of embryo evaluation.

## Competing interests

The authors declare that they have no competing interests.

## Authors’ contributions

GP and CS contribute to the paper by defining the design of the of the study, the analysis and the interpretation of the data. Both authors draft the paper and approved the final version. MS, DV and JVB developed the computer software and added significant information to the critical discussion and approved the final version. SD and TD interpreted the data and revised the paper critically for important intellectual content and approved the final version. All authors read and approved the final manuscript.

## Funding

This study was supported by the Grant for Fertility Innovation 2010(Merck-Serono).
